# MiR-339-5p inhibits breast cancer cell migration and invasion in vitro and may be a potential biomarker for breast cancer prognosis

**DOI:** 10.1186/1471-2407-10-542

**Published:** 2010-10-09

**Authors:** Zheng-sheng Wu, Qiang Wu, Chao-qun Wang, Xiao-nan Wang, Yan Wang, Jing-jing Zhao, Shan-shan Mao, Gui-hong Zhang, Nong Zhang, Xiao-chun Xu

**Affiliations:** 1 Department of Pathology, Anhui Medical University, Hefei, Anhui, People's Republic of China; 2 Department of Pathology, Shanghai Medical College, Fudan University, Shanghai, People's Republic of China; 3 Department of Microbiology and Parasitology, Anhui Medical University, Hefei, Anhui, People's Republic of China; 4 Department of Clinical Cancer Prevention, The University of Texas MD Anderson Cancer Center, Houston, Texas, USA

## Abstract

**Background:**

MicroRNAs (miRNAs) play an important role in the regulation of cell growth, differentiation, apoptosis, and carcinogenesis. Detection of their expression may lead to identifying novel markers for breast cancer.

**Methods:**

We profiled miRNA expression in three breast cancer cell lines (MCF-7, MDA-MB-231, and MDA-MB-468) and then focused on one miRNA, miR-339-5p, for its role in regulation of tumor cell growth, migration, and invasion and target gene expression. We then analyzed miR-339-5p expression in benign and cancerous breast tissue specimens.

**Results:**

A number of miRNAs were differentially expressed in these cancer cell lines. Real-time PCR indicated that miR-339-5p expression was downregulated in the aggressive cell lines MDA-MB-468 and MDA-MB-231 and in breast cancer tissues compared with benign tissues. Transfection of miR-339-5p oligonucleotides reduced cancer cell growth only slightly but significantly decreased tumor cell migration and invasion capacity compared with controls. Real-time PCR analysis showed that BCL-6, a potential target gene of miR-339-5p, was downregulated in MDA-MB-231 cells by miR-339-5p transfection. Furthermore, the reduced miR-339-5p expression was associated with an increase in metastasis to lymph nodes and with high clinical stages. Kaplan-Meier analyses found that the patients with miR-339-5p expression had better overall and relapse-free survivals compared with those without miR-339-5p expression. Cox proportional hazards analyses showed that miR-339-5p expression was an independent prognostic factor for breast cancer patients.

**Conclusions:**

MiR-339-5p may play an important role in breast cancer progression, suggesting that miR-339-5p should be further evaluated as a biomarker for predicting the survival of breast cancer patients.

## Background

Breast cancer is accounted approximately 26% of new cases of all cancers in women in 2009 and is the third most common cause of cancer death in the Unite States [1]. Breast cancer, like other cancers, occurs through multiple genetic alterations, such as activation of oncogenes or inactivation of tumor-suppressor genes. Although improved screening techniques now help detect breast cancer at early stages and advanced treatment remarkably improves patient survival, tumor invasion and metastasis still contribute to the great majority of breast cancer deaths. Our efforts towards the diminution of the disease should include developing novel biomarkers for early detection, prediction of treatment response, monitoring disease progression, and prognosis as well as novel approaches for the treatment and prevention of breast cancer. Although currently there are various techniques for screening breast cancer, such as clinical and self breast examinations, mammography, genetic screening, ultrasound, and magnetic resonance imaging, useful genetic screening biomarkers remain uncommon, and a novel approach is urgently needed.

MicroRNAs (miRNAs), a class of non-protein-coding RNAs, can postranscriptionally silence protein expression either by binding to complementary target messenger RNAs, thereby degrading these messenger RNAs, or by inhibiting them from translating into proteins [2-4]. Recent studies have demonstrated that miRNAs are useful attractive candidates for biomarkers for the early detection and prognosis of breast cancer. For example, miR-21, miR-10b, and miR-335 have been shown to be involved in the formation and progression of breast cancer by targeting certain oncogenes or tumor suppressors [5-7]. Most importantly, a number of studies have found that miRNAs play an important role in the regulation of cell growth, differentiation, apoptosis, and carcinogenesis [8-13].

In this study, we first determined the expression profile of miRNAs using miRNA arrays in different breast cancer cells with diverse invasive ability and then chose one of them, miR-339-5p, for further study of its expression in breast cancer tissue specimens using in situ hybridization. We then explored its role in breast cancer cell growth and invasion capacity as well as its potential for targeting genes. Our goal was to determine whether detection of miRNA expression might be a useful prognostic biomarker in patients with breast cancer.

## Methods

### Cell lines and cultures

The human breast cancer cell lines MCF-7, MDA-MB-231, and MDA-MB-468 were obtained from the American Type Culture Collection (Rockville, MD). MCF-7 cells were grown in RPMI 1640 medium and MDA-MB-231 and MDA-MB-468 in Leibovitz's L-15 cell culture medium with 10% fetal bovine serum at 37°C in a humidified atmosphere of 95% air and 5% CO_2_.

### RNA isolation and miRNA microarray analysis

Total RNA from the breast cancer cell lines or fresh tissue samples was extracted using the mirVANA RNA isolation kit (Ambion, Austin, TX) according to the manufacturer's instructions. After the RNA samples were quantified on a NanoDrop spectrophotometer (Nanodrop, Wilmington, DE), they were stored in aliquots at -80°C until use. For microarray analysis, the RNA samples were first labeled with fluorescence dye hy3 or hy5 using a miRCURY Hy3/Hy5 power labeling kit and then hybridized onto the miRCURY LNA array (both obtained from Exiqon, Copenhagen, Denmark). This miRNA array contained 847 human miRNA sequences based on Sanger miRBase release 11.0. The microarray was scanned using the Axon GenePix 4000B microarray scanner and analyzed using GenePix pro V6.0, and the data were then normalized by the locally weighted scatter plot smoothing (LOWESS) method based on the background-subtracted data. All these procedures were performed by Exiqon.

### Real-time polymerase chain reaction

RNA samples from breast cancer cell lines or tissue samples were subjected to reverse transcription reactions in a mixture of dNTP, a RevertAid MMuLV reverse transcriptase, and RiboLock ribonuclease inhibitor (Applied Biosystems, Foster City, CA). Next, the cDNA was amplified by real-time PCR (ABI PRISM 7300 Sequence Detection System, Applied Biosystems) in an SYBR Green I Real-Time PCR kit (GenePharma, Shanghai, China) for expression of miR-339-5p. U6 small nuclear RNA was used as an internal control. The following primers were used: miR-339-5p, 5'-GTCGTATCCAGTGCGTGTCGTGGAGTCGGCAATTGCACTGGATACGACCGTGAGCTC-3' (a stem-loop primer); 5'-GGGTCCCTGTCCTCCA-3' (forward); 5'-TGCGTGTCGTGGAGTC-3' (reverse) and U6, 5'-GCTTCGGCAGCACATATACTAAAAT-3' (forward); 5'-CGCTTCACGAATTTGCGTGTCAT-3' (reverse). The PCR amplification protocol was as follows: an initial 95°C for 5 min and 50 cycles of 94°C for 15 s, 55°C for 30 s, and 70°C for 30 s. The threshold cycle (Ct) was defined as the fractional cycle number at which the fluorescence passes the fixed threshold. The relative quantification of miRNA expression in each sample was calculated with the 2 ^- ΔΔ Ct ^method after normalization for expression of the positive control. Along with the reverse transcription and real-time PCR, a negative control without Taq enzyme or cDNA template was used to exclude PCR contamination and genomic DNA.

To quantify expression of B-cell lymphoma gene 6 (BCL-6) mRNA, we used the SYBR Premix Ex Taq (Perfect Real Time) kit (TaKaRa, Dalian, China) to detect BCL-6 mRNA levels with the ABI PRISM 7300 sequence detector. Glyceraldehyde-3-phosphate dehydrogenase (GAPDH) mRNA was used as an internal control. The following primers were used: for BCL-6, 5'-CCAGCCACAAGACCGTCCAT-3' (forward) and 5'-CTCCGCAGGTTTCGCATTT-3' (reverse); and for GAPDH, 5'-TGCACCACCAACTGCTTAGC-3' (forward) and 5'-GGCATGGACTGTGGTCATGAG-3' (reverse).

### Transient miRNA transfection

Breast cancer MDA-MB-231 and MCF-7 cells (1.0 × 10^6 ^per well) were seeded and grown overnight in six-well plates. The next day, they were transfected with either miR-339-5p mimic (GenePharma, Shanghai, China), 2'-O methylated single-stranded miR-339-5p antisense oligonucleotides (ASO) (GenePharma, Shanghai, China), or the control oligonucleotides using Lipofectamine 2000 (Invitrogen, Carlsbad, CA) following the manufacturer's instructions. The miRNA mimics are small double-stranded RNA oligonucleotides. The negative control RNA (GenePharma) was used to eliminate the potential nonsequence-specific effects, and the sequences were nonhomologous to any human genome sequences. The sequences of miR-339-5p mimic and ASO were 5'-UCCCUGUCCUCCAGGAGCUCACG UGAGCUCCUGGAGGACAGGGAUU-3' and 5'-CGUGAGCUCCUGGAGGACAGGGA-3', respectively. Nonspecific sequences were 5'-CAGUACUUUUGUGUAGUACAA-3' (a negative control for miRNA mimic), 5'-UUCUCCGAACGUGUCACGUTT-3' (sense), and 5'-ACGUGACACGUUCGUAGAATT-3' (antisense), a negative control for mRNA antisense transfection. Transfection efficiency was confirmed by real-time PCR analysis of miR-339-5p levels in the transfected breast cancer cells.

### Cell viability assay

MCF-7 cells (5 × 10^3^) were suspended in RPMI 1640 medium (100 μl) containing 10% fetal bovine serum and cultured in 96-well plates overnight and then transfected with miR-339-5p ASO or negative control oligonucleotides for 48, 72, and 96 h, respectively. The cell viability was determined by using a WST-8 cell counting Kit-8 (Beyotime, Jiangsu, China). Briefly, CCK-8 solution (10 μl) from the kit was added to each well of the 96-well plates, the plates were incubated at 37°C in a CO_2 _cell incubator for 90 min, and the absorbance rates were measured at 450 nm using a microplate reader (Infinite M200; Tecan, Austria). All experiments were performed in triplicate and repeated trice. The data were plotted as means ± SD of three separate experiments.

### Wound-healing assay

An *in vitro *wound healing/scratch assay was used to assess capacity for tumor cell motility. Briefly, MCF-7 cells (1 × 10^6^/well) were seeded in six-well plates, cultured overnight, and transfected with miR-339-5p ASO and the negative control. Upon reaching confluency, the cell layer was scratched with a sterile plastic tip and then immediately washed with growth medium twice and cultured again in RPMI 1640 medium (including 10% FBS) at 37°C in a humidified incubator with 5% CO_2 _for up to 72 h. At different time points, photo images of the plates were taken under a microscope. The wound healing was measured at 0, 24, 48, and 72 h, and the data were summarized based on sextuple assays for each experiment.

### Cell migration and invasion assay

We used a Transwell insert (24-well insert, pore size 8 μm; Corning, Inc., Corning, NY) to determine the effect of miR-339-5p on breast cancer cell migration and invasion *in vitro*. Briefly, the transfected MDA-MB-231 and MCF-7 cells were first starved in medium without fetal calf serum (FCS) overnight, and the cells (5 × 10^5 ^for MDA-MB-231 and 1 × 10^6 ^for MCF-7) were resuspended in the FCS-free medium and placed in the top chambers in triplicate. The lower chamber was filled with 10% FCS as the chemoattractant and incubated for 36 h (for MDA-MB-231) or 48 h (for MCF-7) for the invasion assay. For the invasion assay, the inserts were previously coated with extracellular matrix gel from Engelbreth-Holm-Swarm mouse sarcoma (BD Biosciences, Bedford, MA). At the end of the experiments, the cells on the upper surface of the membrane were removed using cotton buds, and the cells on the lower surface of the insert were fixed and stained with 0.1% crystal violet. Five visual fields of each insert were randomly chosen and photographed under a light microscope at 200 × magnification. The cells in the photographs were counted, and the data were summarized as means ± standard deviation and presented as a percentage of controls.

### Breast tissue specimens

Ninety consecutive surgical breast tissue specimens from patients with breast cancer and 26 from patients with benign breast disease were obtained from the First Affiliated Hospital of Anhui Medical University (Hefei, Anhui, China) between November 2001 and June 2002. Patients who had received any chemotherapy or radiation therapy before surgery or who had rheumatic disease, acute infection, HIV, or other types of cancer were excluded from the current study. The pathohistological diagnosis of the specimens was consistent with breast neoplasm in accordance with WHO guidelines [14]. Histologic grade was based on the Scarff-Bloom-Richardson system [15]. All breast cancer patients were female and had undergone radical mastectomy or modified radical mastectomy. Of the 90 patients, 86 had full follow-up data. The median follow-up time was 60 months (range 8-64 months).

The protocol for the use of tissue samples from patients and follow-up study was approved by our Institutional Review Board, and every patient had signed a consent form. Our study procedures were in accordance with the ethical standards of the responsible committee on human experimentation and with the Helsinki Declaration of 1975, as revised in 2008.

### Tissue microarray construction

Formalin-fixed and paraffin-embedded tissue blocks of the patients' tissues were retrieved from the Department of Pathology, the First Affiliated Hospital of Anhui Medical University, Hefei, China. Hematoxylin and eosin-stained tissue sections were then reviewed to identify representative tissues for tissue microarray (TMA). Three to five cores (size of 1 mm each) were obtained from the paraffin-embedded blocks for each patient for assembling the TMA blocks by using a manual tissue arrayer (Hengtai Instruments Inc., Liaoning, China). A total of three TMA blocks were prepared and sectioned for in situ hybridization analysis of miRNA expression.

### In situ hybridization

The assay was performed as described previously [16-18] for analysis of miR-339-5p expression in breast tissue specimens. Briefly, 3-μm-thick TMA sections were deparaffinized, rehydrated, and then digested with protease and refixed in 4% paraformaldehyde. After that, the sections were prehybridized with 150 μl of hybridization solution and incubated with LNA-modified probes (Exiqon, Copenhagen, Denmark) for miR-339-5p, U6 (a positive control), or scrambled RNA (a negative control) at 60°C for 20 h. After the sections were washed with 2 × SSC, and then 1 × SCC, they were incubated with a mouse biotinylated anti-digoxigenin antibody followed by incubation with a streptavidin-biotin-peroxidase complex solution. For color reaction, the sections were incubated with a 3,3'-diaminobenzidine solution and counterstained with hematoxylin. The stained sections were reviewed and scored under a light microscope (Olympus America, Melville, NY) independently by two investigators. The staining intensity and percentage of tissue staining were recorded. If less than 10% of the cells stained weakly positive, the core tissue was scored as negative. If more than 10% of the cells stained positive, this core was scored as positive [18]. If any of the three to five cores per case stained positive, that case was considered to be positive

### Bioinformatic and statistical analysis

We searched three miRNA databases (miRbase [19], TargetScan [20], and Pic Tar [21]) for prediction of miR-339-5p targets. All statistical analyses were performed using SPSS software for Windows (version 13.0; SPSS, Chicago, IL). Differences between two groups were compared using Pearson's chi-square test for qualitative variables and Student's t-test for continuous variables. Kaplan-Meier curves were constructed to determine patient relapse-free survival and overall survival rates. The statistical differences in survival among subgroups were compared using the log-rank test. A multivariate Cox proportional hazards regression analysis was used to confirm predictors of case fatality. *P *values < 0.05 were considered statistically significant.

## Results

### Expression of miR-339-5p in breast cancer cells and tissue samples

**Figure 1:**
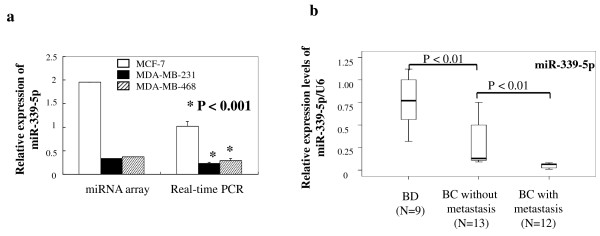
**Expression of miR-339-5p in breast cancer cells and tissues**. *A*. miRNA microarray and real-time PCR analysis of miR-339-5p expression. MDA-MB-231 and MCF-7 cells were grown in monolayer for 5 days, and total RNA was extracted from the cells and subjected to microarray and real-time PCR analyses. *B*. Real-time PCR analysis of miR-339-5p expression. Fresh tissue samples from breast cancer patients with or without lymph node metastasis and from patients with benign breast diseases were obtained from the surgery room. Total RNA was extracted from the tissues and subjected to real-time PCR analysis.

To explore the differential expression of miRNAs in breast cancer cells with different potentials for invasion and metastasis, we performed the miRCURY LNA array analysis in three breast cancer cell lines, MCF-7, MDA-MB-468, and MDA-MB-231 cells. The data were analyzed according to the tumor cell behavior: for example, Group 1 contained low-invasive MCF-7 cells, and Group 2 included relatively highly invasive MDA-MB-231 and MDA-MB-468 cells. The standard of statistical significance was the corrected ratio of hybridization signal intensity between Group 1 (MCF-7 cells) and Group 2 (MDA-MB-231 or MDA-MB-468 cells). If their ratios were equal or more than 2.0 or less than 0.5, expression of miRNAs was considered significantly different. Of the 847 human miRNAs on the array, 98 showed significant differences between Group 1 and Group 2 tumor cells (Additional file 2, Table S1). In particular, 46 mRNAs were upregulated and 52 miRNAs were downregulated (Additional file 1, Fig. S1) in Group 2 cells compared with Group 1 cells, based on hierarchical clustering analysis. We then chose miR-339-5p for further study because its expression was reduced more than fivefold and tenfold in MDA-MB-231 and MDA-MB-468 cells, respectively, compared with MCF-7 cells in the miRNA microarray analysis. Real-time PCR analysis confirmed that miR-339-5p was significantly lower in the MDA-MB-231 and MDA-MB-468 cells than in MCF-7 cells (*P *< 0.001, Fig. 1A).

Next, we used breast tissue specimens to further verify the array data on cell lines. We performed real-time PCR analysis of miR-339-5p expression in fresh tissues from 12 breast cancer patients with lymph node metastasis, 13 breast cancer patients without lymph node metastasis, and nine patients with benign breast disease. We found that levels of miR-339-5p expression in breast cancer with lymph node metastasis were significantly lower than levels for breast cancer without lymph node metastasis or for benign disease (*P *< 0.01, Fig. 1B).

### Effects of miR-339-5p on breast cancer cell growth

**Figure 2:**
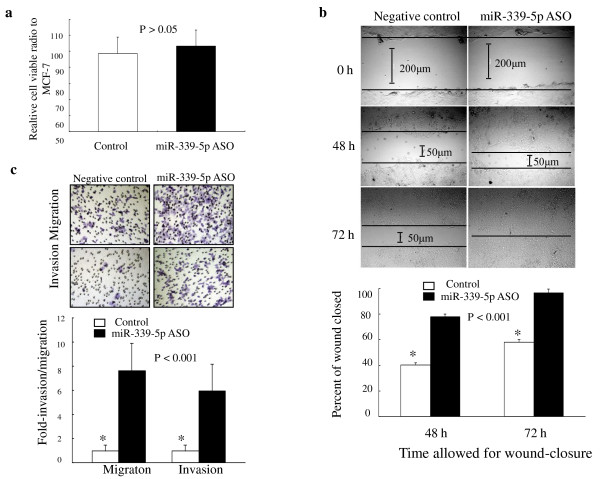
**Effects of miR-339-5p on breast cancer cells**. *A*. Regulation of breast cancer cell growth by miR-339-5p. MCF-7 cells were grown and transiently transfected with miR-339-5p antisense oligonucleotide or scrambled sequence oligonucleotide as negative control for 4 days, and cell viability was analyzed using a kit. The experiments were in triplicate and repeated twice. *B*. Wound-healing assay. MCF-7 cells were grown and transiently transfected with miR-339-5p antisense oligonucleotides or scrambled sequence oligonucleotide as negative control for 3 days and subjected to the wound-healing assay. Magnification: ×100. *C*. Transwell migration and Matrigel invasion assays. MCF-7 cells were grown and transiently transfected with miR-339-5p antisense oligonucleotides or scrambled sequence oligonucleotide as negative control for 2 days and subjected to migration and invasion assays. Representative photographs (upper) and quantification (lower) are shown. Magnification: ×200.

To determine the effects of miR-339-5p in breast cancer cells, we first performed transient miRNA transfection and cell viability assays to detect its ability to regulate tumor cell viability in MCF-7 and MDA-MB-231 cells. We found that knockdown of miR-339-5p expression in MCF-7 cells using miR-339-5p ASO did not significantly reduce the cell viability (Fig. 2A), and expression of miR-339-5p in MDA-MB-231 cells using miR-339-5p mimic oligonucleotides also did not induce tumor cell growth (data not shown).

### Effects of miR-339-5p on breast cancer cell migration and invasion

We next assayed whether miR-339-5p can change the capacity of breast cancer cells for migration and invasion. MDA-MB-231 and MCF-7 cells were selected for expression and knockdown of miR-339-5p using transient gene transfection. As shown in Fig. 2B, tumor cells with miR-339-5p knockdown rapidly closed the scratch wounds compared with the control cells (*P *< 0.001). Moreover, the cell migration and invasion assay showed that miR-339-5p knockdown resulted in increased MCF-7 cell migration rate (7.60 ± 2.28-fold, *P *< 0.001) and invasion rate (5.93 ± 2.23-fold, *P *< 0.001) compared with the control cells (Fig. 2C). In contrast, ectopic expression of miR-339-5p in MDA-MB-231 cells resulted in significant reduction of cell migration and invasion in transwell assays (*P *< 0.001) (data not shown).

### BCL-6, a putative target of miR-339-5p

**Figure 3:**
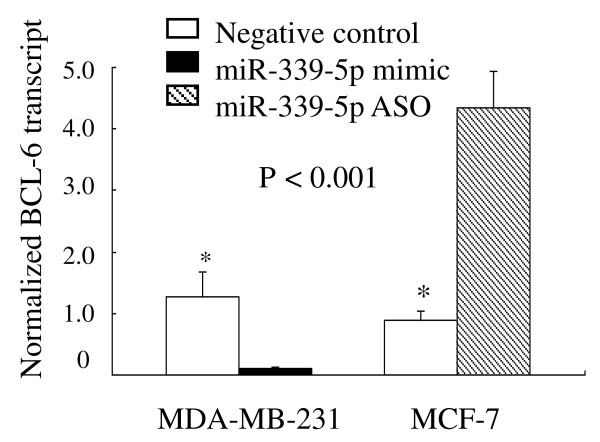
**Regulation of BCL-6 mRNA expression by miR-339-5p in breast cancer cells**. MDA-MB-231 and MCF-7 cells were grown and transiently transfected with miR-339-5p mimic and miR-339-5p ASO, respectively and then subjected to RNA extraction and real-time PCR analysis.

Bioinformatic analyses found that the BCL-6 gene, among a number of others, may be a potential miR-339-5p target. We chose BCL-6 for further analysis because it was predicted by all three algorithms and was also previously implicated in promoting breast cancer progression [22-24]. Indeed, we found that MCF-7 cells transfected with miR-339-5p ASO or MDA-MB-231 cells transfected with miR-339-5p mimics had dramatically increased or lowered BCL-6 levels by 5 fold and 10 fold, respectively, compared with the control cells (both *P *< 0.001, Fig. 3).

### Expression of miR-339-5p in breast tissue specimens and the clinicopathologic features of the patients

**Figure 4:**
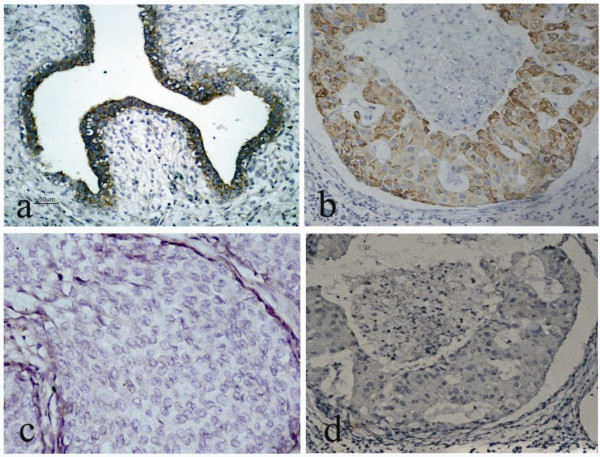
**Expression of miR-339-5p in breast tissue specimens**. Paraffin blocks from breast cancer or benign tissue specimens were prepared for making tissue microarrays, and the sections from the latter were hybridized with miR-339-5p probe in situ hybridization. *A*. Positive expression of miR-339-5p in a breast fibroadenoma. *B*. Positive expression of miR-339-5p in an invasive ductal carcinoma without lymph node metastasis. *C*. Negative expression of miR-339-5p in an invasive ductal carcinoma with lymph node metastasis. *D*. Negative control probe in an invasive ductal carcinoma. Magnification: ×200.

Next, we utilized digoxigenin-labeled locked nucleic acid (LNA)-miRNA probes to detect miR-339-5p levels in archived breast tissue specimens. A scrambled oligonucleotide was used to demonstrate specificity of hybridization. A total of 90 paraffin-embedded tissue specimens of breast cancer, 24 adjacent normal breast and 26 benign breast disease specimens were used, and the data showed that miR-339-5p expression in benign breast tissues was at high levels, with a positive signal predominantly in luminal epithelial cells in both ductual and lobular structures, whereas its expression in breast cancer tissues was lower (*P *= 0.017, Fig. 4 and Table 1).

**Table 1:** Expression of miR-339-5p in cancerous, adjacent normal and benign breast tissues

		miR-339-5p expression	
		
Group	*n*	Negative, *n *(%)	Positive, *n *(%)
Adjacent normal	24	12 (50.0)	12 (50.0)
Benign breast disease	26	11 (42.3)	15 (57.7)
Breast cancer	90	63 (70.0)	27 (30.0)*

Note: * *P *= 0.017.

Futhermore, we associated miR-339-5p expression with the clinicopathologic data from the breast cancer patients and found that miR-339-5p expression was inversely associated with lymph node metastasis (*P *= 0.018) and high clinical stages (*P *= 0.005). However, there was no association between miR-339-5p expression and age, tumor size, tumor grade, c-erbB-2 overexpression, or estrogen receptor and progesterone receptor status (Table 2).

**Table 2:** Association of miR-339-5p expression with clinicopathological data from breast cancer patients

Variable	*n*	MiR-339-5p expression, *n *(%)	*P *value
Age (years)			
≤35	6	0 (0)	0.12
35-55	48	18 (37.5)	
> 55	36	9 (25.0)	
Tumor size (cm)			
≤2	4	2 (50.0)	0.67
2-5	68	20 (29.4)	
> 5	18	5 (27.8)	
Lymph node metastases			
0	29	14 (48.3)	0.018
1-3	33	9 (27.3)	
> 3	28	4 (14.3)	
Grade			
I	4	1 (25.0)	0.17
II	57	21 (36.8)	
III	29	5 (17.2)	
Stage			0.005
I - II	50	21 (42.0)	
III - IV	40	6 (15.0)	
Estrogen receptor			
-	57	18 (36.1)	0.67
+	33	9 (27.3)	
Progesterone receptor			
-	56	16 (28.6)	0.70
+	34	11 (32.4)	
c-erbB-2 expression			
Low	59	18 (30.5)	0.89
High	31	9 (29.0)	

### Association of miR-339-5p expression with overall survival and relapse-free survival

**Figure 5:**
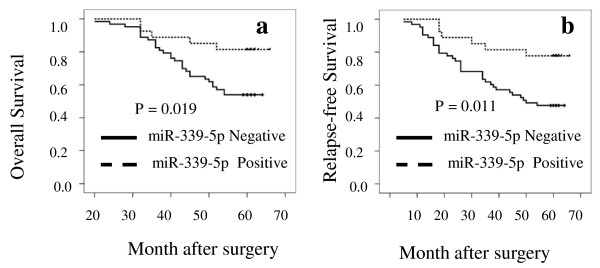
**Association of miR-339-5p expression with overall survival and relapse-free survival of the patients with breast cancer**. *A*, Overall survival (OS). *B*, Relapse-free survival (RFS).

We then performed Kaplan-Meier analyses to determine whether miR-339-5p expression was associated with overall survival and relapse-free survival of the breast cancer patients. Patients whose primary tumors did not express miR-339-5p (n = 63) had a mean 5-year overall survival of 53.0 months (54%), whereas patients with tumors expressing miR-339-5p (n = 27) had a mean overall survival of 61.0 months (82%). The difference was statistically significant (*P *= 0.019, Fig. 5). Similarly, a statistically significant association of miR-339-5p with relapse-free survival was also found (*P *= 0.011).

In addition, Cox proportional hazards model analyses showed that miR-339-5p expression was an independent prognostic factor for 5-year survival of patients with breast cancer (hazard ratio = 0.38; 95% CI: 0.16-0.92; *P *= 0.032).

## Discussion

In the current study, our data showed that a number of miRNAs were differentially expressed in these cancer cell lines. One of them was miR-339-5p, which had a downregulated expression in the more aggressive breast cancer cell lines and tissue samples. Our further study showed that miR-339-5p could significantly decrease tumor cell migration and invasion capacity, which associated with downregulation of BCL-6 expression in breast cancer cells. *In ex vivo*, miR-339-5p expression was lower in breast cancer tissues than in benign breast tissues, which was associated with an increase in tumor metastasis to lymph nodes and with high clinical stages, and tumors expressing miR-339-5p had better overall and relapse-free survival rates compared with those without miR-339-5p expression. Thus, miR-339-5p expression was an independent prognostic factor for these patients. The data from the current study suggest that miR-339-5p may play an important role in breast cancer development and progression and that miR-339-5p should be further evaluated as a biomarker for predicting patient survival.

Indeed, recent studies have reported altered expression of miR-339-5p in several kinds of solid tumors, including gastric cancer, lung cancer, and cervical cancer [25-27]. Tie et al. analyzed the global miRNA expression profile in highly invasive and low-invasive gastric cancer cells using a miRNA microarray and found that miR-339-5p was significantly downregulated in highly invasive gastric cancer cells [25]. These data are consistent with our current results for breast cancer cells. However, these former studies did not pursue further investigation on miR-339-5p.

We therefore further explored the role of miR-339-5p in breast cancer cell lines. MCF-7, MDA-MB-231, and MDA-MB-468 cells have very different capacities for invasive and metastatic potentials *in vivo*. The differential expression of miR-339-5p in these cell lines suggests that miR-339-5p may play a role in regulation of breast cancer cell migration and invasion. Our current findings showed that expression of miR-339-5p did reduce migration and invasion capacity of MDA-MB-231 cells, whereas knockdown of miR-339-5p expression in MCF-7 increased migration and invasion of the cells. In contrast, miR-339-5p showed limited ability to affect breast cancer cell growth.

In our bioinformatic analyses to search for potential target genes of miR-339-5p, we focused our attention on BCL-6, because BCL-6 contains the binding sites of miR-339-5p in its 3'-UTR. The human proto-oncogene BCL-6 encodes a BTB/POZ-zinc finger transcriptional repressor that was originally characterized as a regulator of B-lymphocyte development and growth and has been implicated in the pathogenesis of B cell lymphoma [28,29] and in cancers of many organs, including breast cancer [30,22-24]. For example, Bajalica et al. reported that BCL-6 was expressed in mammary glands of nonpregnant animals and during early pregnancy and that BCL-6 could prevent ductal formation and apoptosis [30]. BCL-6 was found to be overexpressed in breast cancer [22] and in 68% of histological high-grade ductal breast cancers, clinically the most aggressive subgroup of breast cancer. BCL-6 was able to prevent mammary epithelial differentiation [23]. Furthermore, Pinto et al. [24] demonstrated that BCL-6 could significantly increase expression of three metastasis-related genes -- CXC-chemokine receptor 4 (CXCR4) [31], fms-like tyrosine kinase-1 (FLT-1) [32], and integrin-b-3 [33] -- in breast cancer cell lines. Our current study revealed that miR-339-5p could inhibit expression of BCL-6 mRNA, which is associated with suppression of the migration and invasion of breast cancer cells. However, it is not clear whether inhibition of BCL-6 expression by miR-339-5p is direct or indirect; further studies are needed for clarification.

Our current study also found that lost expression of miR-339-5p was associated with breast cancer metastasis to lymph nodes, higher clinical stages, poor overall survival, and poor relapse-free survival of breast cancer patients. To our knowledge, this is the first study of miR-339-5p in breast cancer, although a number of other miRNAs have been reported to be altered in breast cancer. For example, Scott and colleagues reported that miR-125a and miR-125b could reduce invasion and migration capacity of SKBR3 breast cancer cells through targeting ERBB2 and ERBB3 genes [34]. A recent study by Wu et al. using breast cancer tissues and cells demonstrated the important role of miR-205 in breast cancer progression. They found that miR-205 was highly expressed in normal breast tissues and nonmalignant breast epithelial cell lines and that ectopic expression of miR-205 significantly reduced breast cancer cell proliferation, invasion, and metastasis *in vivo *[35]. In addition, two independent studies [7,36] showed that expressions of miR-335, miR-206, miR-126, and miR-146 were significantly decreased in highly invasive breast cancer cell lines, which is consistent with our microarray data presented here.

## Conclusions

This study suggests that miR-339-5p may play an important role in breast cancer progression. However, future study will be needed to verify the usefulness of miR-339-5p as a biomarker in other populations before miR-339-5p can be used in the clinic.

## Competing interests

The authors declare that they have no competing interests.

## Authors' contributions

WZS, WCQ, WXN, WY, ZJJ and MSS performed experiments; WZS, WQ, ZN and XXC designed research and wrote the paper; WZS and ZGH analyzed data. All authors read and approved the final Manuscript.

## Pre-publication history

The pre-publication history for this paper can be accessed here:

http://www.biomedcentral.com/1471-2407/10/542/prepub

## Supplementary Material

Additional file 1 **Unsupervised hierarchical clustering of miRNAs among three breast cell lines**. 52 human miRNAs were downregulated between MCF-7 and MDA-MB-468, or MDA-MB-231 cells. Click here for file

Additional file 2 **Differential expression of miRNAs among three breast cancer cell lines**. 98 human miRNAs showed significant differences between MCF-7 and MDA-MB-468, or MDA-MB-231 cells. Click here for file
