# Supplementary figures and images for: MiR-339-5p inhibits breast cancer cell migration and invasion in vitro and may be a potential biomarker for breast cancer prognosis
Source: BMC Cancer. 2010 Oct 9;10:542. doi: 10.1186/1471-2407-10-542 (PMC2958952; doi:10.1186/1471-2407-10-542)

## Slide 1
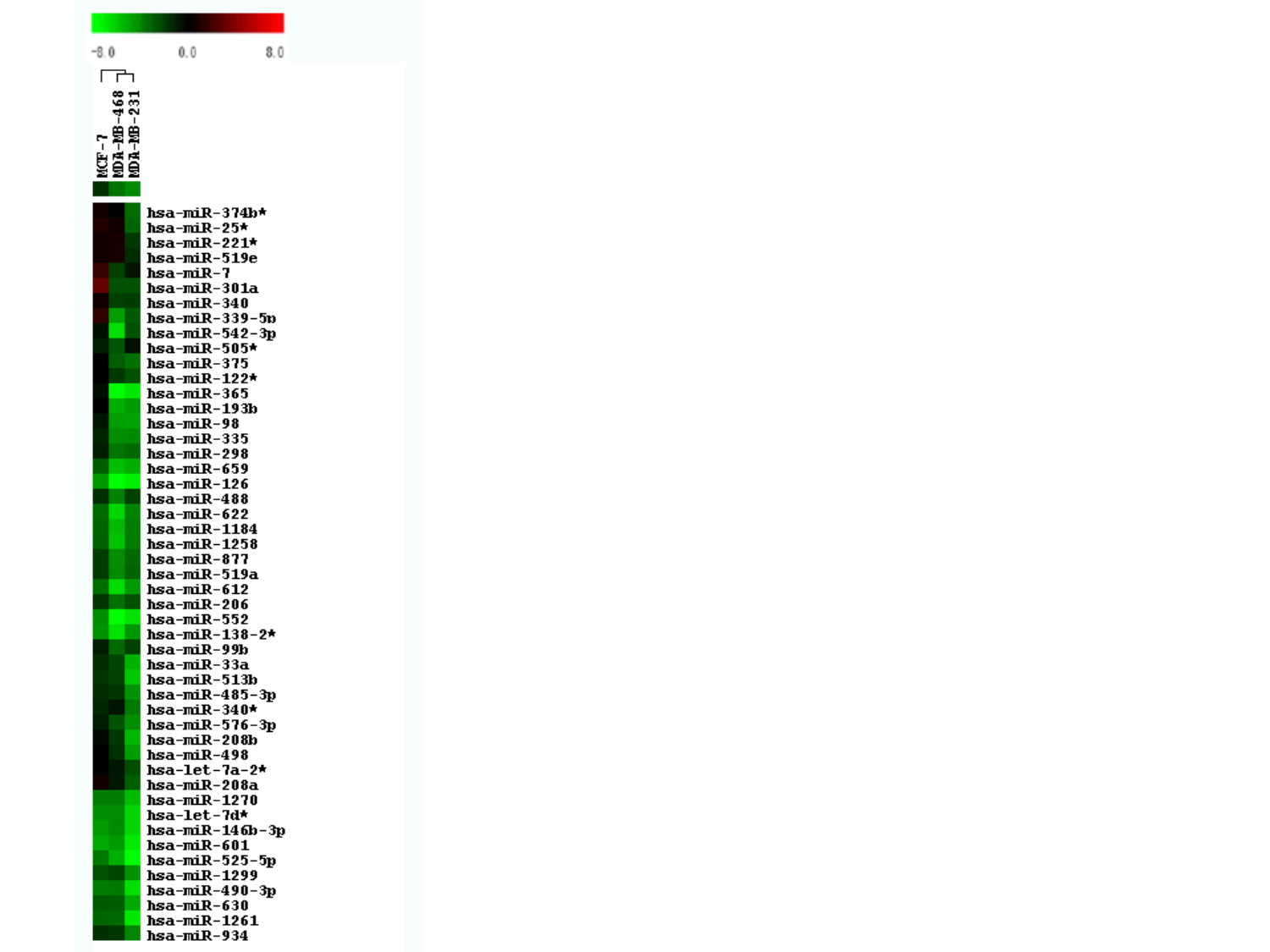

Supplement: Additional file 1 — Unsupervised hierarchical clustering of miRNAs among three breast cell lines. 52 human miRNAs were downregulated between MCF-7 and MDA-MB-468, or MDA-MB-231 cells. [file 1471-2407-10-542-S1.PPT]
