# Supplementary material for: MiR-339-5p inhibits breast cancer cell migration and invasion in vitro and may be a potential biomarker for breast cancer prognosis
Source: BMC Cancer. 2010 Oct 9;10:542. doi: 10.1186/1471-2407-10-542 (PMC2958952; doi:10.1186/1471-2407-10-542)
Supplement: Additional file 2 — Differential expression of miRNAs among three breast cancer cell lines. 98 human miRNAs showed significant differences between MCF-7 and MDA-MB-468, or MDA-MB-231 cells. [file 1471-2407-10-542-S2.DOC]

| **MiRNA** | **MCF-7** | **MDA-MB-231** | **MDA-MB-468** | **Fold change  (231 vs. MCF-7)** | **Fold change (468 vs. MCF-7)** |
| --- | --- | --- | --- | --- | --- |
| hsa-miR-99b | 0.69 | 0.46 | 0.28 | 0.67 | 0.41 |
| hsa-miR-206 | 0.52 | 0.37 | 0.26 | 0.72 | 0.49 |
| hsa-miR-505* | 0.71 | 0.8 | 0.36 | 1.12 | 0.5 |
| hsa-miR-519a | 0.48 | 0.3 | 0.2 | 0.61 | 0.4 |
| hsa-miR-488 | 0.54 | 0.45 | 0.21 | 0.84 | 0.39 |
| hsa-miR-1258 | 0.3 | 0.22 | 0.1 | 0.71 | 0.32 |
| hsa-miR-138-2* | 0.17 | 0.16 | 0.07 | 0.94 | 0.39 |
| hsa-miR-1184 | 0.3 | 0.22 | 0.11 | 0.73 | 0.36 |
| hsa-miR-96 | 0.99 | 0.20 | 0.23 | 0.20 | 0.23 |
| hsa-miR-622 | 0.28 | 0.21 | 0.08 | 0.75 | 0.28 |
| hsa-miR-612 | 0.27 | 0.16 | 0.07 | 0.57 | 0.27 |
| hsa-miR-877 | 0.47 | 0.28 | 0.18 | 0.61 | 0.38 |
| hsa-miR-126 | 0.17 | 0.06 | 0.04 | 0.34 | 0.27 |
| hsa-miR-630 | 0.34 | 0.13 | 0.32 | 0.38 | 0.96 |
| hsa-miR-485-3p | 0.58 | 0.17 | 0.53 | 0.3 | 0.91 |
| hsa-miR-490-3p | 0.22 | 0.07 | 0.23 | 0.32 | 1.05 |
| hsa-miR-25* | 1.48 | 0.29 | 1.22 | 0.19 | 0.82 |
| hsa-let-7a-2* | 0.98 | 0.39 | 0.73 | 0.4 | 0.75 |
| hsa-miR-183 | 3.45 | 0.57 | 0.52 | 0.17 | 0.15 |
| hsa-miR-182 | 0.63 | 0.23 | 0.10 | 0.37 | 0.16 |
| hsa-miR-208a | 1.22 | 0.32 | 0.72 | 0.26 | 0.59 |
| hsa-miR-122* | 0.98 | 0.37 | 0.5 | 0.38 | 0.51 |
| hsa-miR-208b | 0.9 | 0.11 | 0.5 | 0.12 | 0.55 |
| hsa-let-7d* | 0.18 | 0.08 | 0.19 | 0.42 | 1.06 |
| hsa-miR-1299 | 0.37 | 0.17 | 0.43 | 0.47 | 1.17 |
| hsa-miR-1270 | 0.22 | 0.11 | 0.22 | 0.49 | 1.01 |
| hsa-miR-552 | 0.18 | 0.07 | 0.01 | 0.37 | 0.07 |
| hsa-miR-298 | 0.69 | 0.29 | 0.24 | 0.42 | 0.35 |
| hsa-miR-934 | 0.54 | 0.2 | 0.51 | 0.37 | 0.94 |
| hsa-miR-498 | 0.96 | 0.15 | 0.56 | 0.16 | 0.59 |
| hsa-miR-1261 | 0.29 | 0.06 | 0.29 | 0.22 | 1 |
| hsa-miR-301a | 3.38 | 0.37 | 0.37 | 0.11 | 0.11 |
| hsa-miR-33a | 0.57 | 0.12 | 0.42 | 0.21 | 0.75 |
| hsa-miR-519e | 1.26 | 0.57 | 1.34 | 0.45 | 1.06 |
| hsa-miR-375 | 1 | 0.25 | 0.33 | 0.25 | 0.33 |
| hsa-miR-193b | 1.01 | 0.16 | 0.12 | 0.16 | 0.12 |
| hsa-miR-525-5p | 0.23 | 0.04 | 0.12 | 0.18 | 0.54 |
| hsa-miR-98 | 0.76 | 0.14 | 0.15 | 0.19 | 0.2 |
| hsa-miR-339-5p | 1.95 | 0.34 | 0.15 | 0.17 | 0.08 |
| hsa-miR-576-3p | 0.7 | 0.18 | 0.37 | 0.26 | 0.53 |
| hsa-miR-374b* | 1.3 | 0.27 | 0.92 | 0.2 | 0.7 |
| hsa-miR-513b | 0.52 | 0.09 | 0.46 | 0.17 | 0.88 |
| hsa-miR-601 | 0.13 | 0.06 | 0.16 | 0.48 | 1.24 |
| hsa-miR-146b-3p | 0.16 | 0.08 | 0.19 | 0.48 | 1.2 |
| hsa-miR-340* | 0.61 | 0.23 | 0.77 | 0.38 | 1.27 |
| hsa-miR-221* | 1.31 | 0.51 | 1.45 | 0.39 | 1.11 |
| hsa-miR-659 | 0.32 | 0.12 | 0.1 | 0.38 | 0.32 |
| hsa-miR-542-3p | 0.8 | 0.37 | 0.07 | 0.46 | 0.09 |
| hsa-miR-335 | 0.63 | 0.19 | 0.18 | 0.3 | 0.29 |
| hsa-miR-365 | 0.84 | 0.06 | 0.05 | 0.08 | 0.05 |
| hsa-miR-7 | 1.98 | 0.77 | 0.46 | 0.39 | 0.23 |
| hsa-miR-340 | 1.27 | 0.46 | 0.46 | 0.36 | 0.36 |
| hsa-miR-22 | 1.77 | 6.99 | 5.47 | 3.94 | 3.08 |
| hsa-miR-151-5p | 0.20 | 0.66 | 0.63 | 3.24 | 3.08 |
| hsa-miR-28-5p | 0.07 | 0.26 | 0.22 | 3.56 | 2.97 |
| hsa-miR-744 | 0.10 | 0.28 | 0.21 | 2.89 | 2.22 |
| hsa-let-7i | 0.66 | 2.33 | 2.02 | 3.51 | 3.05 |
| hsa-miR-186 | 0.08 | 0.21 | 0.20 | 2.69 | 2.53 |
| hsa-miR-140-3p | 0.01 | 0.21 | 0.23 | 16.23 | 18.27 |
| hsa-miR-140-5p | 0.02 | 0.25 | 0.24 | 12.23 | 11.58 |
| hsa-miR-30e* | 0.08 | 0.21 | 0.20 | 2.55 | 2.48 |
| hsa-miR-23b | 2.11 | 10.65 | 7.22 | 5.05 | 3.43 |
| hsa-miR-1290 | 4.75 | 18.80 | 9.57 | 3.96 | 2.01 |
| hsa-miR-17* | 0.04 | 0.21 | 0.40 | 4.74 | 9.25 |
| hsa-miR-30a | 2.83 | 6.68 | 6.53 | 2.36 | 2.31 |
| hsa-miR-106a | 0.89 | 2.30 | 2.85 | 2.60 | 3.21 |
| hsa-miR-18a | 0.38 | 0.77 | 1.15 | 2.01 | 2.99 |
| hsa-miR-1201 | 0.14 | 0.38 | 0.53 | 2.79 | 3.90 |
| hsa-miR-29b | 0.26 | 1.75 | 1.81 | 6.66 | 6.91 |
| hsa-miR-574-3p | 0.04 | 0.08 | 0.13 | 2.07 | 3.39 |
| hsa-miR-18b | 0.29 | 0.52 | 0.82 | 1.80 | 2.86 |
| hsa-let-7g | 0.90 | 2.11 | 2.65 | 2.33 | 2.93 |
| hsa-let-7d | 0.48 | 1.25 | 1.05 | 2.61 | 2.20 |
| hsa-miR-29a | 0.86 | 26.58 | 25.46 | 30.91 | 29.61 |
| hsa-miR-17 | 1.29 | 3.61 | 4.58 | 2.79 | 3.54 |
| hsa-miR-27a | 0.31 | 1.14 | 1.00 | 3.71 | 3.26 |
| hsa-miR-23a | 2.34 | 6.88 | 5.87 | 2.94 | 2.51 |
| hsa-let-7a | 1.54 | 5.80 | 4.68 | 3.76 | 3.03 |
| hsa-miR-1259 | 3.43 | 9.69 | 7.71 | 2.83 | 2.25 |
| hsa-miR-151-3p | 0.16 | 0.53 | 0.50 | 3.30 | 3.10 |
| hsa-miR-20b* | 0.43 | 0.83 | 0.93 | 1.91 | 2.15 |
| hsa-miR-129-5p | 0.09 | 0.33 | 0.27 | 3.48 | 2.83 |
| hsa-let-7f | 4.04 | 8.63 | 8.64 | 2.14 | 2.14 |
| hsa-miR-19b | 0.62 | 1.62 | 1.96 | 2.61 | 3.16 |
| hsa-miR-574-5p | 0.13 | 0.16 | 0.37 | 1.26 | 2.87 |
| hsa-miR-20a | 0.40 | 1.26 | 1.86 | 3.13 | 4.64 |
| hsa-miR-125b-1* | 0.08 | 0.22 | 0.17 | 2.77 | 2.19 |
| hsa-miR-19a | 1.76 | 4.88 | 5.45 | 2.77 | 3.09 |
| hsa-miR-24 | 4.40 | 15.03 | 10.92 | 3.41 | 2.48 |
| hsa-miR-125b | 0.25 | 14.85 | 14.70 | 58.58 | 58.00 |
| hsa-let-7b | 1.24 | 3.35 | 2.03 | 2.70 | 1.64 |
| hsa-miR-196a* | 0.53 | 1.39 | 0.31 | 2.61 | 0.58 |
| hsa-miR-22* | 0.26 | 0.91 | 0.27 | 3.53 | 1.06 |
| hsa-miR-27b* | 0.03 | 0.15 | 0.09 | 4.50 | 2.65 |
| hsa-miR-30d | 0.14 | 0.28 | 0.21 | 2.04 | 1.55 |
| hsa-miR-320a | 0.45 | 0.99 | 0.87 | 2.18 | 1.93 |
| hsa-miR-320c | 0.41 | 0.90 | 0.81 | 2.18 | 1.96 |
| hsa-miR-422a | 0.05 | 0.17 | 0.03 | 3.13 | 0.46 |
